# Bioinformatics analysis of proteomics profiles in senescent human primary proximal tubule epithelial cells

**DOI:** 10.1186/s12882-016-0249-z

**Published:** 2016-04-01

**Authors:** Yang Lu, Jingchao Wang, Chen Dapeng, Di Wu, Guangyan Cai, Xiangmei Chen

**Affiliations:** Department of Nephrology, Chinese PLA General Hospital, Chinese PLA Institute of Nephrology, Beijing Key Laboratory of Kidney Disease, State Key Laboratory of Kidney Diseases, National Clinical Research Center for Kidney Diseases, General Hospital of PLA, Fuxing Road 28, Beijing, 100853 P.R. China; Department of Nephrology, China-Japan Friendship Hospital, Beijing, China

**Keywords:** Proximal tubule cells, Senescence, Proteomics, Network

## Abstract

**Background:**

Dysfunction of renal tubule epithelial cells is associated with renal tubulointerstitial fibrosis. Exploration of the proteomic profiles of senesced tubule epithelial cells is essential to elucidate the mechanism of tubulointerstitium development.

**Methods:**

Primary human proximal tubule epithelial cells from passage 3 (P3) and passage 6 (P6) were selected for evaluation. EdU and SA-*β-galactosidase* staining were used to detect cell senescence. p53, p21, and p16 were detected by Western blot analysis. Liquid chromatography mass spectrometry (LC-MS) was used to examine differentially expressed proteins (DEPs) between P6 and P3 cells. The expression of DEPs was examined by Western blot analysis. Bioinformatics analysis was performed by protein-protein interaction and gene ontology analyses.

**Results:**

The majority of tubule cells from passage 6 (P6) stained positive for SA-*β-galactosidase*, whereas passage 3 (P3) cells were negative. Senescence biomarkers, including p53, p21, and p16, were upregulated in P6 cells relative to P3 cells. EdU staining results showed a lower rate of EdU positive cells in P6 cells than in P3 cells. LC-MS was used to examine DEPs between P6 and P3 cells. These DEPs are involved in glycolysis, response to stress, cytoskeleton regulation, oxidative reduction, ATP binding, and oxidative stress. Using Western blot analysis, we validated the down-regulation of AKR1B1, EEF2, EEF1A1, and HSP90 and the up-regulation of VIM in P6 cells seen in the LC-MS data. More importantly, we built the molecular network based on biological functions and protein-protein interactions and found that the DEPs are involved in translation elongation, stress, and glycolysis, and that they are all associated with cytoskeleton regulation, which regulates senescent cell activities such as apoptosis and EMT in tubule epithelial cells.

**Conclusions:**

We explored proteomic profile changes in cell culture-induced senescent cells and built senescence-associated molecular networks, which will help to elucidate the mechanisms of senescence in human proximal tubule epithelial cells.

## Background

Aging and aging-related diseases are associated with various health problems worldwide. Renal aging begins around approximately 40 years old and is accompanied by decreased renal blood flow (~10 % per year). The decline in renal function and susceptibility to age-related renal insufficiency may contribute to chronic progressive kidney failure. Senescence of tubule epithelial cells involves multiple complex activities including apoptosis, cell shape enlargement, decreased motility, aberrant energy metabolism, and epithelial-to-mesenchymal transition (EMT), and it is the major cause of renal tubulointerstitial fibrosis [1] and kidney failure. Exploring the mechanism of senescent tubule epithelial cell activities will aid us in understanding the pathogenesis of renal PTEC senescence.

The known senescence-associated cell activities are mainly based on changes in protein components. It has been reported that the upregulation of nuclear factor kappa B (NF-κB), tumor growth factor beta (TGF-β) and hypoxia-inducible factor (HIF) affect tubule epithelial cell proliferation, cellular apoptosis and EMT, which is associated with senescence in tubule epithelial cells [2, 3]. However, there is still a lack of large-scale proteomic analyses that have explored aging-related proteins and mechanisms. Therefore, we performed label-free quantitative proteomics and explored proteomic profiles in senescent human proximal tubule epithelial cells (PTECs) to identify the molecular mechanism underlying senescence-associated cell activities in PTECs.

## Methods

### Isolation and culture of human primary tubular cells

Segments of macroscopically and histologically normal renal cortex were obtained under aseptic conditions from patients undergoing nephrectomy for small (<6 cm) tumors in the Department of Urology, Chinese PLA General Hospital. Patients were accepted into the study if they had no history of renal or systemic disease associated with tubulointerstitial pathology. Tubular fragments were derived from the segments of renal cortex by collagenase digestion and were isolated by centrifugation in 45 % Percoll (Pharmacia, Uppsala, Sweden). The PTECs were re-suspended in a 1:1 (vol/vol) mixture of Dulbecco’s modified Eagle’s (GIBCO^TM^ Invitrogen, Barcelona, Spain) and Ham’s F-12 media (Hyclone, USA) supplemented with 10 % heat-inactivated fetal bovine serum (FBS) (GIBCO^TM^ Invitrogen, Barcelona, Spain), 10 ng/ml EGF (Peprotech, Rocky Hill, USA), 5 mg/ml human transferrin, 5 mg/ml bovine insulin (all from Sigma, St. Louis, MO, USA), 100 U/ml penicillin, and 100 μg/ml streptomycin (Invitrogen, New York, NY, USA). Passage 3 was defined as the young control, and passage 6 was defined as cellular senescence.

### Immunofluorescence

The PTEC biomarker cytokeratin 18 was detected by immunofluorescence. Cells were fixed in 4 % paraformaldehyde and permeabilized with 1 % Triton X-100 buffer. Cells were then incubated with anti-CK18 antibody (Zhongshan Golden Bridge Bio-technology, Beijing, China) and DAPI (Sigma-Aldrich, St. Louis, MO, USA) for nuclear staining. Cells were examined using a Nikon fluorescence microscope (Japan).

### SA-β-gal staining

Cells were fixed with 2 % formaldehyde and 0.2 % glutaraldehyde for 15 min and stained with freshly prepared senescence-associated β-galactosidase (SA-β-gal) (1 mg/mL X-gal, 40 mM citric acid/sodium phosphate (pH 6.0), 5 mM potassium ferrocyanide, 5 mM potassium ferricyanide, 150 mM NaCl, and 2 mM MgCl_2_) overnight at 37 °C without CO_2_. The cells were then examined under a microscope.

### 5-ethynyl-2′-deoxyuridine (EdU) proliferation assay

Proliferative activity was detected using an EdU labeling kit (Roche Ltd, USA) following the manufacturer’s recommendations. Fluorescent images were obtained by florescence microscopy.

### Western blot analysis

Antibodies against p53 (Abcam, Cambridge, UK), p53, AKR1B1, EEF1A1, EEF2, HSP90 (Proteintech Group Inc.), p21, and p16 (Cell Signaling Technology, Danvers MA, USA) were used for Western blot analysis. β-Actin (Sigma-Aldrich, St. Louis, MO, USA) served as a control. Approximately 30 μg of protein were subjected to 12 % sodium dodecyl sulfate-polyacrylamide gel electrophoresis (SDS-PAGE). After incubation in primary and secondary antibodies, images were acquired using an Opti-Chemi 600 (UVP Inc., Upland, CA, USA).

### Label-free quantitative proteomics

Protein (50 μg) was separated by 12 % SDS-PAGE. Gels were stained with R250 Coomassie Brilliant Blue. Each lane of the gel was cut into four fragments, and each fragment was trypsin-digested as described previously [4]. Peptides were analyzed using two-dimensional (2-D) liquid chromatography mass spectrometry (LC-MS) (XEVO QTOF, Waters Corp., Manchester, UK). Samples were separated on a 180-μm × 50-mm Symmetry C18 5 μm (Waters Corp., Manchester, UK) reversed-phase trap column in the first dimension with Solvent A (200 mM ammonium formate, pH 10.0) and solvent B (CH3CN). Five different solvent plugs set automatically by Masslynx 4.1 were applied to elute the fractions sequentially. In the second dimension, peptides were eluted with a nanoACQUITY system equipped with a C18 column (75 μm × 100 mm; Waters Corp.) with solvent A (water) and solvent B (CH3CN). The procedure and data analysis were similar to those in our previous study [5]. The column temperature was maintained at 35 °C. Two hundred femtomolesl/μL of [Glu1] fibrinopeptide B was applied as the lock mass with a constant flow rate of 300 nl/min. Each sample was detected in triplicate. The spectral acquisition time in each mode was 0.6 s. In the low energy MS mode, data were collected at a constant collision energy of 6 eV. In the elevated energy MS mode, the collision energy was increased from 15 to 36 eV. Each sample group contained three replicates that were combined for expression profile analysis by PLGS 2.4. The precursor and fragment ion tolerance were determined automatically. The default protein identification criteria included a maximal protein mass of 500,000 Da and a detection of at least three fragment ions per peptide, seven fragment ions per protein, and one peptide per protein. Fixed modification of carbamidomethyl-C and the detected variable modifications, including acetylation (N-terminus), deamidation (N/Q) and oxidation of methionines, were selected. At most, two missed cleavages and a false positive rate of 4 % were allowed. Normalization was performed using the auto-normalization function, [6] which exhibited an effect similar to the internal standard [7]. The NCBI human database (released in March, 2012) was used as a reference database. Only those proteins identified in at least two of three injections and demonstrating fold changes >1.5 were considered differentially expressed proteins (DEPs).

### Bioinformatics analysis

Data analysis was performed using MAS 3.0 (http://bioinfo.capitalbio.com/mas3/), BiNGO and 2.44 STRING 9.0 (http://string.embl-heidelberg.de) software. The ClueGo and BiNGO 2.44 software and plug-ins for Cytoscape 2.7 were used to analyze the biological functions. String and the MAS 3.0 system were used for protein-protein interaction (PPI) analysis (score >0.6) and PPI network building. Cytoscape 2.7 was used to modify the network.

### Ethics statement

The study protocol was approved by the Ethics Committee of PLA general hospital of China. Written informed consent was obtained from all study participants. The diagnosis of renal cancer was made based on results of renal imaging testing and pathological examination.

## Results

### Tubule cells from passage 6 exhibit an obvious senescence phenotype

**Fig. 1:**
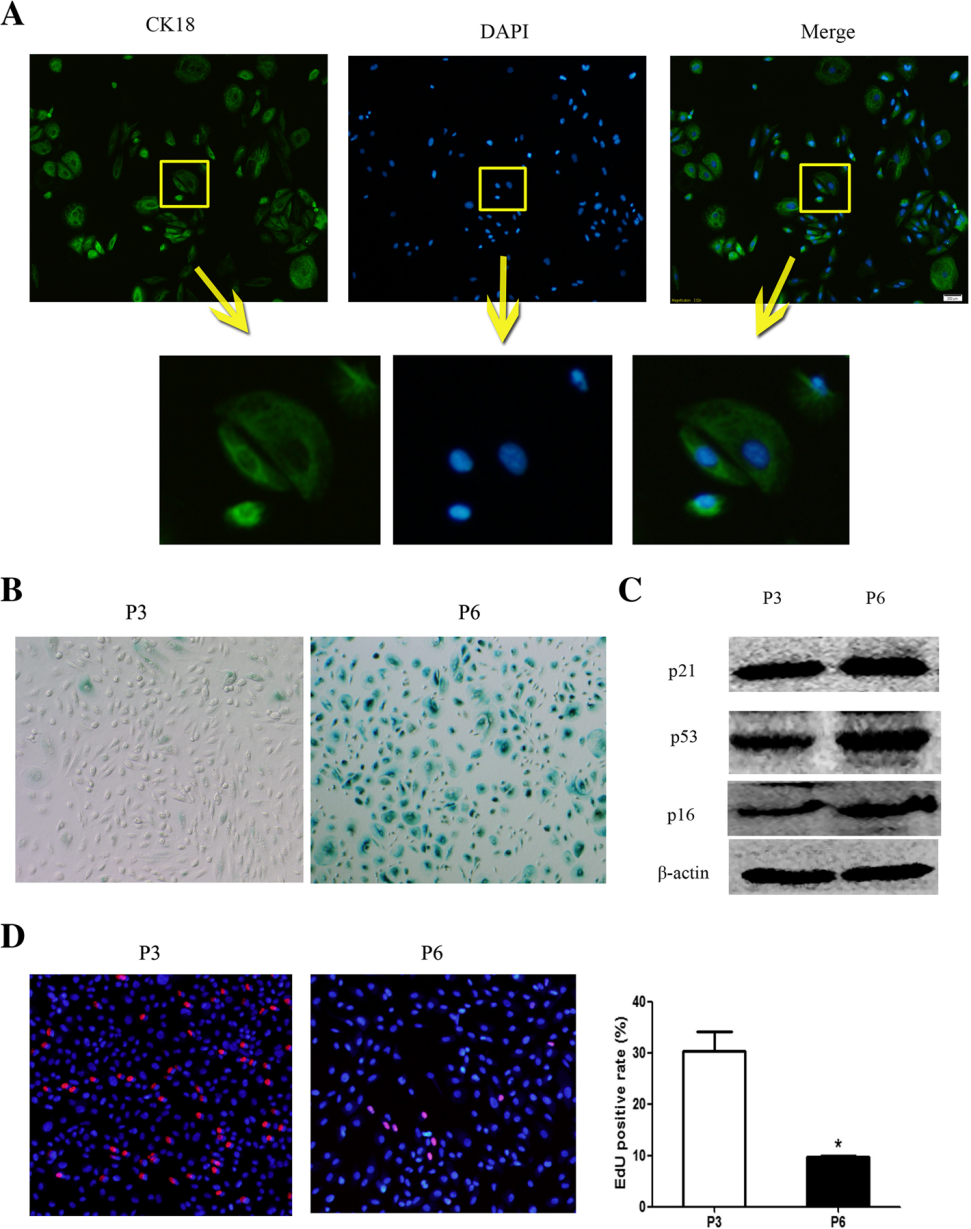
Tubule cells from passage 6 exhibit an obvious senescence phenotype. **a** Primary PTEC was identified by immunofluorescence of CK18. **b** β-galactosidase staining was performed to detect senescence. Nearly all tubule cells from the passage 6 (P6) group stained positive for β-galactosidase. **c** Senescence biomarkers including p53, p21, and p16 were detected by Western blot and upregulated in the P6 group compared to P3 cells. **d** EdU staining results showed that there was lower positive rate in the P6 group than P3 group

We first identified primary proximal tubule cells by confirming the expression of cytokeratin 18 (CK18). Immunofluorescence of CK18 in the cytoplasm confirmed the purity of tubule cells (Fig. 1a). Next, SA-β-gal staining was performed to detect senescence. Nearly all tubule cells from the passage 6 (P6) group stained positive for SA-β-gal (Fig. 1b). Moreover, the expression of senescence biomarkers including p53, p21, and p16 were detected and upregulated in the P6 group compared to passage 3 (P3) cells (Fig. 1c). EdU staining revealed that a lower rate of positivity in P6 cells compared with P3 cells (Fig. 1d). Therefore, P6 tubule cells were defined as senescent.

### LC-MS results showed that DEPs in the senescent PTECs were associated mainly with metabolism, cytoskeleton regulation, oxidative reduction, and stress

**Fig. 2:**
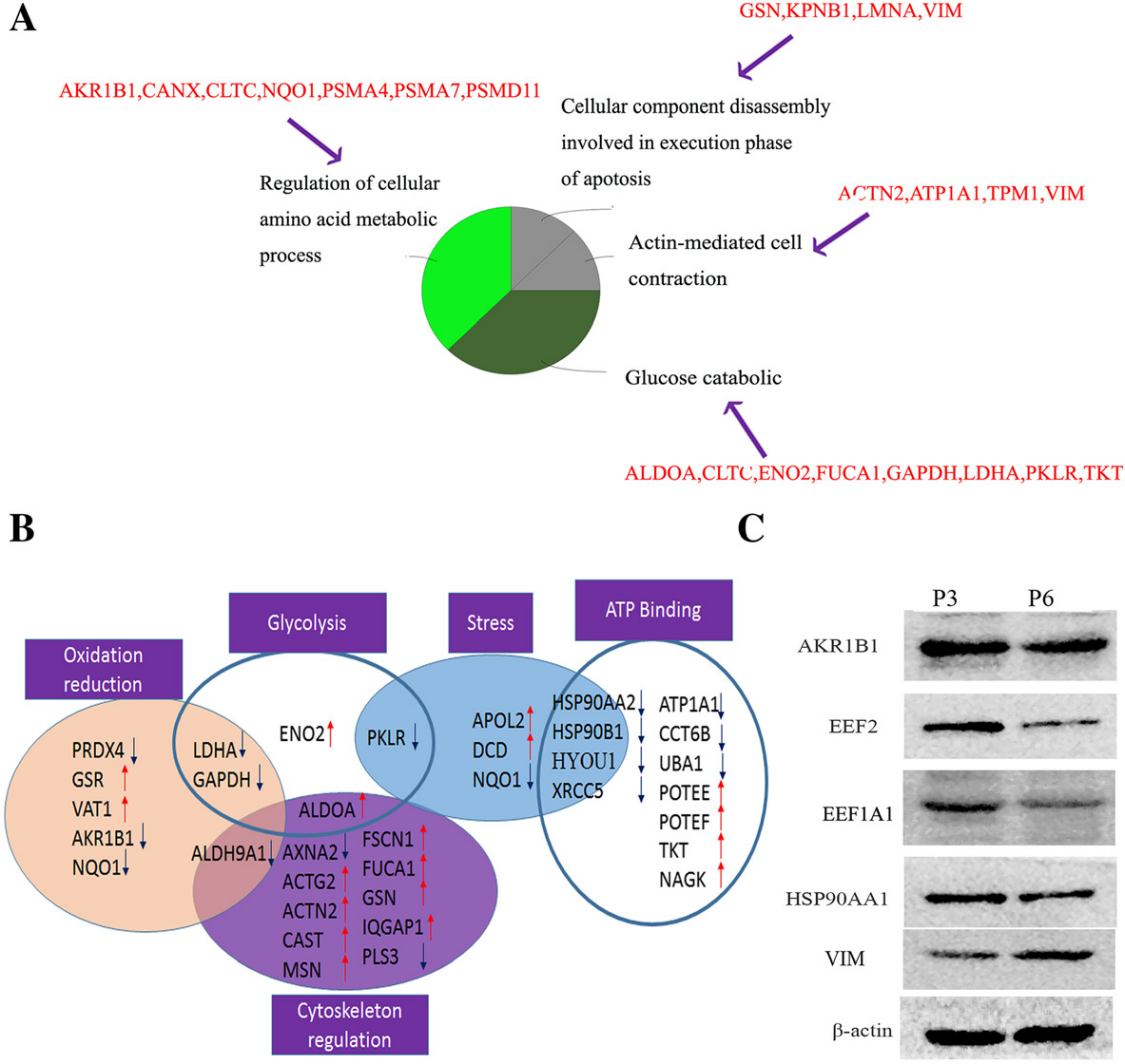
Biological function analysis for proteomics profiles in P6 group. **a** CLUEGO analysis showed the DEPs in P6 group are involved in the regulation of cellular amino acid metabolic processes, apoptosis, actin-mediated cell contraction, and glucose catabolism. **b** BinGO analysis revealed additional biological functions, including glycolysis, response to stress, cytoskeleton regulation, oxidative reduction, adenosine triphosphate (ATP) binding, and oxidative stress. **c** the expression DEPs including AKR1B1, EEF2, EEF1A1 and HSP90 in the network was validated by western blot (*Red up arrow* meant DEPs upregulated in P6, and *blue down arrow* meant DEPs downregulated in P6)

LC-MS was used to examine the DEPs between P3 and P6 renal tubule epithelial cells. Thirty-four proteins were downregulated and 36 proteins were upregulated in P6 cells compared to P3 cells (Tables 1 and 2). We then applied two tools to analyze the functions of these DEPs. CLUEGO analysis showed that these proteins are involved in the regulation of cellular amino acid metabolic processes, apoptosis, actin-mediated cell contraction, and glucose catabolism (Fig. 2a). BinGO analysis revealed additional biological functions, including glycolysis, response to stress, cytoskeleton regulation, oxidative reduction, adenosine triphosphate (ATP) binding, and oxidative stress (Fig. 2b). These DEPs regulate biological functions related to the process of senescence in PTECs. Moreover, we also validated the expression of DEPs by Western blot analysis (Fig. 2c). We confirmed that AKR1B1, EEF2, EEF1A1, and HSP90 were downregulated and that VIM was upregulated in P6 cells, which is consistent with our proteomic results.

**Table 1:** The downregulated DEPs in P6 tubule cells detected by LC-MS (fold change >1.5)

Accession	Description	Protein	P6/P3 ratio	Standard deviation
NP_001074007.2	aldo keto reductase family 1 member B15	AKR1B15	0.01	0.006
NP_001002858.1	annexin A2	ANXA2	0.12	0.025
NP_001619.1	aldose reductase	AKR1B1	0.17	0.026
XP_933678.1	PREDICTED POTE ankyrin domain family member I	POTEI	0.18	0.15
NP_002037.2	glyceraldehyde 3 phosphate dehydrogenase	GAPDH	0.30	0.03
NP_000289.1	pyruvate kinase isozymes R L	PKLR	0.37	0.016
NP_003290.1	endoplasmin precursor	HSP90B1	0.41	0.006
NP_002256.2	importin subunit beta 1	KPNB1	0.41	0.02
NP_003325.2	ubiquitin like modifier activating enzyme 1	UBA1	0.43	0.007
NP_005339.3	heat shock protein HSP 90 alpha	HSP90AA2	0.44	0.004
NP_001153706.1	sodium potassium transporting ATPase subunit alpha 1	ATP1A1	0.48	0.019
NP_001171651.1	glucose 6 phosphate isomerase	GPI	0.61	0.009
NP_001393.1	elongation factor 1 alpha 1	EEF1A1	0.66	0.009
NP_945189.1	protein glutamine gamma glutamyltransferase 2	TGM2	0.63	0.017
NP_065843.3	neutral cholesterol ester hydrolase 1	AADACL1	P3	
NP_000687.3	4 trimethylaminobutyraldehyde dehydrogenase	ALDH9A1	P3	
NP_004299.1	rho GTPase activating protein 1	ARHGAP1	P3	
NP_001719.2	basigin precursor	BSG	P3	
NP_001019820.1	calnexin precursor	CANX	P3	
NP_006575.2	T complex protein 1 subunit zeta 2	CCT6B	P3	
NP_002942.2	dolichyl diphosphooligosaccharide protein glycosyltransferase subunit 2 precursor	DDOST	P3	
NP_004721.1	eukaryotic peptide chain release factor subunit 1	ETF1	P3	
NP_110416.1	minor histocompatibility antigen H13	HM13	P3	
NP_006380.1	hypoxia up regulated protein 1 precursor	HYOU1	P3	
NP_005557.1	L lactate dehydrogenase A chain	LDHA	P3	
NP_001244303.1	lamin	LMNA	P3	
NP_000894.1	NADPH dehydrogenase quinone 1	NQO1	P3	
NP_005023.2	plastin 3	PLS3	P3	
NP_006397.1	peroxiredoxin 4 precursor	PRDX4	P3	
NP_001096137.1	proteasome subunit alpha type 4	PSMA4	P3	
NP_653263.2	proteasome subunit alpha type 7 like	PSMA7	P3	
NP_055205.2	staphylococcal nuclease domain containing protein 1	SND1	P3	
NP_110437.2	thioredoxin domain containing protein 5 precursor	TXNDC5	P3	
NP_066964.1	X ray repair cross complementing protein 5	XRCC5	P3

**Table 2:** The upregulated DEPs in P6 tubule cells detected by LC-MS (Fold change >1.5)

Accession	Description	Protein	P6/P3 ratio	Standard deviation
NP_001182032.1	glutathione reductase mitochondrial isoform 3 precursor	GSR	2.03	0.18
NP_001605.1	actin cytoplasmic 2	ACTG1	3.22	0.05
NP_001966.1	gamma enolase	ENO2	3.74	0.45
NP_001093241.1	POTE ankyrin domain family member F	POTEF	3.82	0.064
NP_001077007.1	POTE ankyrin domain family member E	POTEE	3.90	0.065
NP_006363.4	heterogeneous nuclear ribonucleoprotein Q	SYNCRIP	4.10	0.27
NP_005991.1	tubulin alpha 4A chain	TUBA4A	4.85	0.12
NP_001094.1	alpha actinin 2	ACTN2	p6	
NP_001121089.1	fructose bisphosphate aldolase A	ALDOA	p6	
NP_112092.1	apolipoprotein L2	APOL2	p6	
NP_006076.4	3 2 5 bisphosphate nucleotidase 1	BPNT1	p6	
NP_775083.1	calpastatin	CAST	p6	
NP_004850.1	clathrin heavy chain 1	CLTC	p6	
NP_000080.2	collagen alpha 2 I chain precursor	COL1A2	p6	
NP_444513.1	dermcidin preproprotein	DCD	p6	
NP_004238.3	116 kDa U5 small nuclear ribonucleoprotein component	EFTUD2	p6	
NP_001129490.1	epoxide hydrolase 1 precursor	EPHX1	p6	
NP_003079.1	fascin	FSCN1	p6	
NP_000138.2	tissue alpha L fucosidase precursor	FUCA1	p6	
NP_000168.1	gelsolin precursor	GSN	p6	
NP_003861.1	ras GTPase activating like protein	IQGAP1	p6	
NP_002435.1	moesin	MSN	p6	
NP_038479.1	myoferlin	MYOF	p6	
NP_060037.3	N acetyl D glucosamine kinase	NAGK	p6	
NP_002769.1	proactivator polypeptide preproprotein	PSAP	p6	
NP_002806.2	26S proteasome non ATPase regulatory subunit 11	PSMD11	p6	
NP_055113.2	nicotinate nucleotide pyrophosphorylase carboxylating precursor	QPRT	p6	
NP_001003.1	40S ribosomal protein S8	RPS8	p6	
NP_056456.1	testin	TES	p6	
NP_001055.1	transketolase	TKT	p6	
NP_001018005.1	tropomyosin alpha 1 chain	TPM1	p6	
NP_005992.1	tubulin alpha 3C D chain	TUBA3D	p6	
NP_997195.1	tubulin alpha 3E chain	TUBA3E	p6	
NP_006364.2	synaptic vesicle membrane protein VAT 1 homolog	VAT1	p6	
NP_003371.2	vimentin	VIM	p6	
NP_001152994.1	putative zinc finger protein 727	ZNF727	p6

### Biological functions, including translation elongation, stress, and glycolysis, were all associated with cytoskeleton regulation based on PPI in senescence-associated molecular networks

**Fig. 3:**
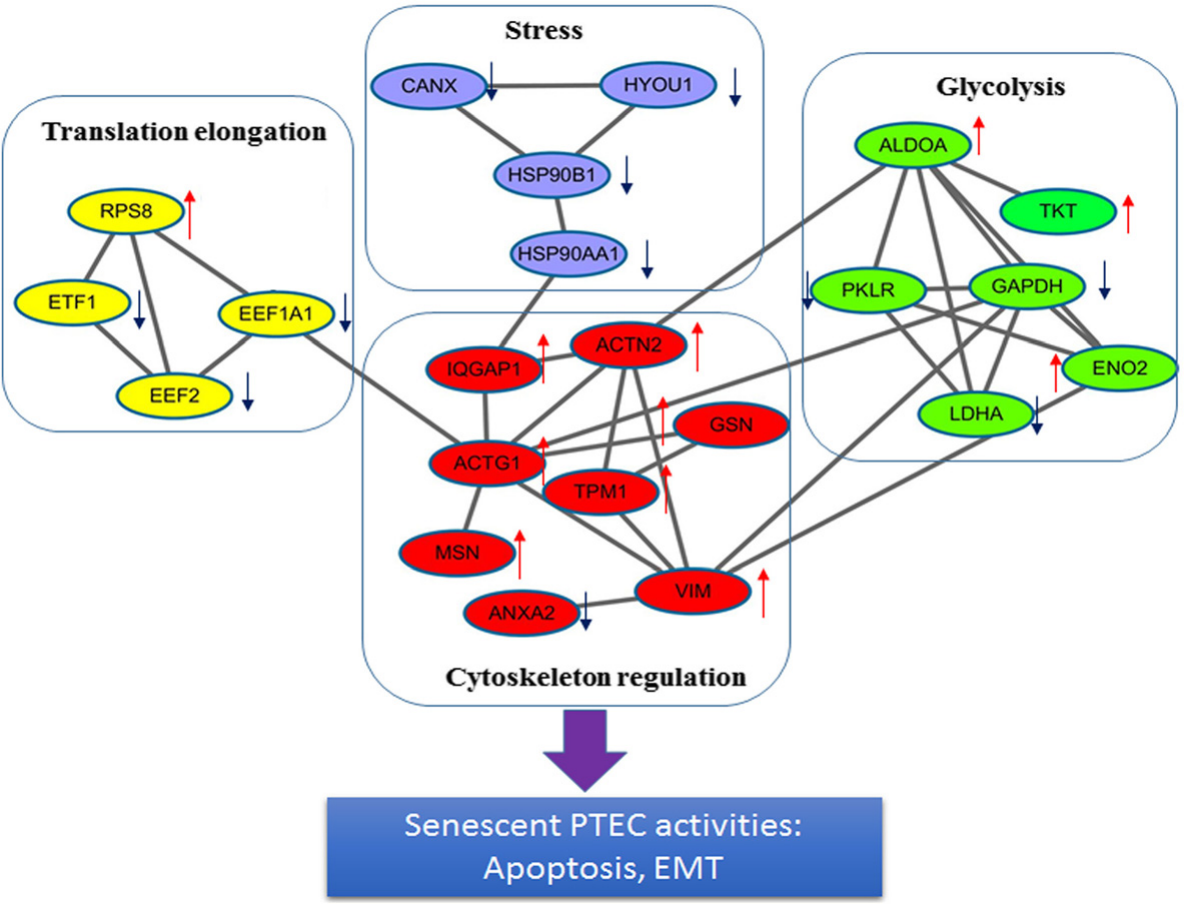
Biological functions including translation elongation, stress and glycolysis could mediate the senescence-cell activities by acting on cytoskeleton regulation. The molecular network of DEPs was built by protein-protein interactions. In the network, RPS8, ETF1, EEF1A1, and EEF2 regulate eukaryotic translation elongation (Color: *yellow*), TKT, GAPDH, ALDOA, ENO2, LDHA, and PKLR are involved in glycolysis (Color: *blue*), DEPs, such as ACTN2, VIM, ANXA2, MSN, and GSM mediate cytoskeleton regulation (Color: *red*). CANX, HSP90B1, HSP90AA1 and HYOU1 are associated with oxidative stress (Color: *green*). Translation elongation, stress, glycolysis were all act on the cytoskeleton regulation, and in turn regulate cell activities in senescent PTEC (*Red up arrow* meant DEPs upregulated in P6, and *blue down arrow* meant DEPs downregulated in P6)

To better explore the mechanisms involved in PTEC senescence, we built molecular networks based on PPI (Fig. 3). In the network, EEF1A1 and EEF2 regulate eukaryotic translation elongation, and GAPDH, ALDOA, ENO2, LDHA, and PKLR are involved in glycolysis. Other DEPs, such as ACTN2, VIM, ANXA2, MSN, and GSM mediate cytoskeleton regulation. HSP90B1, HSP90AA1 and HYOU1 are associated with oxidative stress. More importantly, translation elongation, stress and glycolysis were all related to cytoskeleton regulation, which was associated with regulation of PTEC apoptosis and EMT.

## Discussion

In this study, the specific protein changes involved in human PTEC senescence were explored. The altered proteins were found to be involved in regulating senescence-associated biological functions including cytoskeleton regulation, glycolysis, stress and metabolism. More importantly, these biological functions can affect each other via PPI, which provide new insights on the mechanism of senescence in PTECs.

Aberrant energy metabolism, such as glucose hysteresis, is an important cause of aging. In this study, we determined that key enzymes such as PKLR, ALDOA, GAPDH, and LDHA were disrupted and contributed to a disturbance in glycolysis. ALDOA is a key enzyme that catalyzes the reversible conversion of fructose-1,6-bisphosphate to glyceraldehydes-3-phosphate (GAPDH) and dihydroxyacetone phosphate in glycolysis [8]. LDHA catalyzes the interconversion of pyruvate and lactate PKLR, and, as a pyruvate kinase, catalyzes the transphosphorylation of phosphoenolpyruvate into pyruvate and ATP, which is the final step of glycolysis [9]. In P6 cells, low-expression of LDHA, GAPDH and PKLR may be involved in reduced glycolytic function, which could subsequently promote glucose hysteresis. Renal tubule epithelial cells are high-energy-demanding polarized epithelial cells [10]. In diabetic patients, senescent tubule epithelial cells may be prone to glucose metabolic dysfunction under hyper-glucose conditions.

Stress plays a crucial role in senescence. In our study, we revealed that most stress-associated proteins including HYOU1, HSP90, NQO1, and XRCC were downregulated in aging renal tubule epithelial cells. Specifically, HYOU1 is associated with endoplasmic reticulum stress [11], HSP90 is a stress-induced protein that participates in stress resistance [12], NQO1 protects against oxidative stress induced by a variety of metabolic situations, and XRCC5 (Ku80) is crucial for stress-induced DNA double-strand break repair [13]. These proteins may play a crucial role in mediating stress in aging renal tubule epithelial cells.

Eukaryotic translation elongation factors are also closely related to senescence. It was reported that EEF1A1 and EEF1B2 could serve as senescence-associated biomarkers, which are downregulated during cellular senescence [14]. In this study, we confirmed that EEF1A1 and EEF2 were downregulated in senescent renal tubule cells (see Fig. 3). EEF1A1 is one of the alpha subunit forms of the elongation factor 1 complex that interacts with aminoacylated tRNA and facilitates its delivery to the ‘A’ site of the ribosome during the elongation phase of protein synthesis. EEL1A1 is involved in moonlighting functions, including cytoskeletal remodeling, protein folding and degradation, cell signaling modulation, control of cell growth, apoptosis, and cell cycle. Therefore, our results also suggest that EEF1A1 may serve as a biomarker of renal tubule epithelial cell senescence.

More importantly, we explored molecular networks to define the role of biological functions in PTECs. In the network, translation elongation, stress, and glycolysis were associated with cytoskeleton regulation by PPI. We found that DEPs mediating cytoskeleton regulation were closely associated with regulating cell activities such as EMT and apoptosis in aging PTECs. For example, upregulation of DEPs VIM, IQGAP1, and moesin is closely related to EMT and renal fibrosis, [15, 16] and GSN, another DEP, is related to renal tubule epithelial cell apoptosis [17]. We deduced that translation elongation, stress, and glycolysis may regulate senescent cell activities such as apoptosis and EMT by influencing cytoskeleton regulation in PTECs [18–20]. EMT is a common change in cell phenotype of renal tubule epithelial cells, especially in those cells undergoing senescence. However, in this study, although P6 cells showed EMT-like characteristics (vimentin upregulation and E-cadherin downregulation), most cells maintained an epithelial cell morphology with CK18 expression. This result is supported by other reports, which showed that PTECs underwent EMT upon chemokine (ex. TGF-β) stimulation [21–23]. We deduce that most P6 cells cannot undergo EMT without a cytokine stimulus. This may help to explain why those exhibiting senescence in their kidneys may show greater renal fibrosis in the event of inflammation or nephrology.

## Conclusions

We identified specific proteomic profiles involved in cell culture-induced senescence of renal tubule epithelial cells and built a senescence-associated biological function network involved in regulation of PTEC senescence activities. These results will aid in understanding the mechanisms involved in renal tubule epithelial cell senescence.

## Abbreviations

LC-MS: liquid Chromatograph Mass Spectrometer
EMT: epithelial-to-mesenchymal transition
PTEC: proximal tubule epithelial cells
DEP: differentially expressed protein
LDHA: L lactate dehydrogenase A chain
HYOU1: hypoxia up regulated protein 1 precursor
GAPDH: glyceraldehyde 3 phosphate dehydrogenase
PKLR: pyruvate kinase isozymes R L
LDHA: L lactate dehydrogenase A chain
HSP90B1: endoplasmin precursor
NQO1: NADPH dehydrogenase quinone 1
EEF1A1: elongation factor 1 alpha 1
VIM: vimentin
IQGAP1: ras GTPase activating like protein
GSN: gelsolin precursor
